# Type II Activation of Macrophages and Microglia by Immune Complexes Enhances Th17 Biasing in an IL-6-Independent Manner

**DOI:** 10.1371/journal.pone.0164454

**Published:** 2016-10-12

**Authors:** Sarrabeth Stone, Anne Camille La Flamme

**Affiliations:** 1 School of Biological Sciences, Victoria University of Wellington, Wellington, New Zealand; 2 Malaghan Institute of Medical Research, Wellington, New Zealand; Universitatsklinikum Freiburg, GERMANY

## Abstract

Macrophages can be activated into several distinct activation states. One of these states, type II activation, has a regulatory phenotype characterized by decreased IL-12 and increased IL-10, and has been shown to bias naïve CD4^+^ T cells to a Th2 response. Microglia, the resident macrophage-like cells in the central nervous system (CNS), are important contributors to neuroinflammation and, thus, we investigated if type II activated microglia could bias CD4^+^ T cell responses in a similar manner as type II activated macrophages. Using immune complex ligation in the presence of LPS to induce type II activation, we found that both type II macrophages and type II microglia biased CD4^+^ T cell responses *in vitro* to express increased levels of IL-17A and CD124. The enhanced IL-17A production occurred independently of IL-6, and IL-10 and IL-12, which were key regulators of IFN-γ production, but were not involved in the increased IL-17A. Finally, we found that another type II-activating compound, glatiramer acetate, did not bias CD4^+^ T cells to produce enhanced IL-17A. Taken together, this study demonstrates that microglia can be type II activated and, similarly to type II macrophages, can bias CD4^+^ T cell responses; however, depending on the type II stimulus, the effect on CD4^+^ T cell subset differentiation may vary.

## Introduction

Macrophages are capable of being activated into several different forms through exposure to various environmental stimuli [1, 2]. While this activation is generally considered to occur on a spectrum, several distinct activation states have been identified, including classical (M1), alternative (M2a) and type II or regulatory macrophages (M2b) [1, 2]. Classical macrophages are generated through exposure to LPS following IFN-γ priming, and are proinflammatory, producing high levels of IL-12 and co-stimulatory molecule expression. Conversely, type II macrophages (generated through stimulation with LPS and immune complexes, IC) produce higher levels of IL-10, and lower levels of IL-12 and several co-stimulatory/inhibitory molecules such as CD40, CD86, CD80 and PD-L1 [3]. Previous studies have also shown that while classical macrophages bias naïve CD4^+^ T cells toward a Th1 phenotype, type II macrophages drive CD4^+^ T cells towards a Th2 response *in vitro* [4] and *in vivo* [3]. However, assessment of the ability of type II macrophages to bias T cell responses has been largely limited to the Th1/Th2 dichotomy [5], and the factors involved in the biasing of T cell responses have not been fully investigated.

Microglia are cells of the myeloid lineage and are the only resident immune cells in the CNS. Thus, they are considered to be very important in the initiation and development of the immune responses in the CNS; however, depending on the situation, microglia may play either a pathological or a protective role in neuroinflammation. For example, when IL-23 and CD40 are not expressed by cells in the CNS, the severity of disease is decreased in experimental autoimmune encephalomyelitis (EAE) an animal model of multiple sclerosis (MS) [6, 7]. Furthermore, when the Th2 cytokine IL-4 is not expressed by resident CNS cells the severity of EAE is increased [8]. Thus, microglia can affect the type of immune response that develops in the CNS [6] and this regulation may depend upon the activation state of the microglia.

The current study aimed to understand more fully how type II activation of macrophages and microglia modified T cell responses. To this end we investigated T cell biasing beyond the Th1/Th2 dichotomy and dissected the pathways involved in this biasing by type II macrophages and microglia.

## Materials and Methods

### Mice

C57BL/6 mice were bred at the Malaghan Institute of Medical Research (Wellington, New Zealand). 2D2 mice, which express a transgenic T cell receptor (TCR) specific for myelin-oligodendrocyte glycoprotein (MOG_35-55_), were bred at Victoria University of Wellington (Wellington, New Zealand). Mice were housed with access to food and water *ad libitum* and were monitored daily for any physical signs of disease or discomfort. The use of healthy mice as a source of primary immune cells (bone marrow-derived macrophages, microglia, and CD4 T cells) was approved by the Animal Ethics Committee of Victoria University of Wellington (2011-R21).

### Bone marrow macrophage derivation

Bone marrow macrophages were derived using GM-CSF and IL-3 as described in *Current Protocols in Immunology* [9]. Briefly, progenitor cells (pooled from 1–2 mice per experiment) were harvested from tibias and femurs of 8–16 week old C57BL/6 mice with Dulbecco’s phosphate buffered saline (dPBS, Life Technologies, Carlsbad, CA, USA) containing 100 U/ml penicillin plus 100 mg/ml streptomycin (Life Technologies), and red blood cells (RBC) were lysed with RBC lysis buffer (Sigma, St Louis, MO USA). Cells (1x10^6^/ml) were cultured overnight in complete medium containing Dulbecco’s modified essential medium, 10% FCS, 100 U/ml penicillin plus 100 mg/ml streptomycin, 10 mM Hepes, 2 mM L-glutamine and 50 μM 2-mercaptoethanol (all from Life Technologies). 2-mercaptoethanol, a reducing agent, was used to prevent the accumulation of free oxygen radicals. The non-adherent population was then isolated and cultured in the presence of GM-CSF and IL-3 (both at 5 ng/ml, Peprotech, Rocky Hill, NJ USA) for 9 days with fresh media containing GM-CSF and IL-3 (final concentration of both 2.5 ng/ml) added on the 5^th^ day in culture. The adherent population (i.e. macrophages) was removed by pipetting following incubation with ice cold dPBS (Ca^2+^ and Mg^2+^-free; Life Technologies).

### *In vitro* macrophage and T cell co-cultures

Bone marrow macrophages (1x10^5^ cells/well) were cultured in complete medium in 96-well, round bottomed plates (BD Biosciences, Franklin Lakes, NJ, USA) and primed overnight in the presence of IFN-γ (20 U/ml; Peprotech). For macrophage:T cell co-culture, IFN-γ was washed out with warm media before macrophages were stimulated with or without LPS (200 ng/ml, Sigma) in the presence or absence of IC (10 IC per macrophage, 1x10^6^/well). IC were generated from fresh sheep RBC (SRBC; isolated from a healthy sheep, Taylor Prestons Ltd. Wellington, New Zealand) opsonised with rabbit anti-SRBC IgG polyclonal antibody (Sigma). Briefly IC were generated by incubating SRBC with a non-agglutinating concentration of anti-SRBC IgG in dPBS (Life Technologies) for 30 minutes at room temperature and centrifuged at 300 x g for 5 minutes. The supernatant was aspirated to remove non-bound antibody and the IC were resuspended in complete media.

Spleens (pooled from 1–2 mice per experiment) from 2D2 mice were dissociated by passing them through a 70 μM cell strainer (BD Biosciences). RBCs were lysed with RBC lysis buffer (Sigma) and CD4^+^ T cells were isolated using CD4 (L3T4) Dynabeads (Life Technologies) according to manufactures instructions. Four hours after the addition of LPS and/or IC to macrophage cultures, 2D2 CD4^+^ T cells (2.5 x 10^5^ cells/well) were added to the macrophage cultures along with the MOG_35-55_ peptide (25 μg/ml; Genescript). Neutralising antibodies (all from BD Biosciences) and recombinant cytokines (all from BD Biosciences) were added at the time of macrophage stimulation or four hours post stimulation as indicated.

### Carboxyfluorescein succinimidyl ester (CFSE) dye dilution assay

CD4^+^ T cells (pooled from 1–2 mice per experiment) were isolated from 2D2 mice as described above and resuspended at 2x10^7^ cells/ml in dPBS (Life Technologies). 625 nM CFSE (Molecular Probes, Life Technologies) was added and incubated at room temperature for 8 minutes in the dark before the reaction was quenched by adding an equal volume of 100% FCS. Cells were washed once with dPBS (Life Technologies) and twice with complete medium Dulbecco’s modified essential medium, 10% FCS, 100 U/ml penicillin plus 100 mg/ml streptomycin, 10 mM Hepes, 2 mM L-glutamine and 50 μM 2-mercaptoethanol (all from Life Technologies), before being used in T cell co-cultures. For some experiments, 1 mM aminoguanidine hemisulfate (Sigma) was added to the T cell co-cultures to inhibit iNOS. Cell proliferation was assessed by flow cytometry using a Canto II flow cytometer (BD Biosciences)

### Microglia culture and microglia-T cell co-culture

Microglia (pooled from 5 mice per experiment) were derived as described previously [10]. Briefly 4–6 week old mice were euthanised, perfused with heparinised PBS (Sigma; 1 U/ml), and CNS tissue was removed. A single cell suspension of CNS tissue was generated using a 70 μm cell strainer and resuspended in 10 ml 70% Percoll^™^ (Sigma), which was overlaid with equal volumes of 37% and 30% Percoll^™^. This gradient was centrifuged at 760 x g for 30 minutes with no brake, and the microglia were isolated from the 70:37% interface. Microglia purity was assessed by flow cytometry (CD45^lo^, CD11b^+^; mean purity = 83.2 ± 1.6% from 26 experiments). Microglia were seeded at 5x10^4^ cells/well in microglia media (Dulbecco’s modified essential medium, 10% FCS, 100 U/ml penicillin plus 100 mg/ml streptomycin, 45 μM 2-mercaptoethanol; all from Life Technologies, and 10 ng/ml M-CSF (R&D systems, Minneapolis, MN, USA) in a flat-bottomed 96-well plate and cultured for four weeks. After 4 weeks in culture, microglia cultures were found to contain primarily mature microglia (Figure A in S1 File). The mature, adherent microglia in the 96-well plates were primed with IFN-γ overnight (20 U/ml), before stimulation with or without LPS (200 ng/ml) and/or IC (1x10^6^/well). For microglia T cell co-culture, IFN-γ was removed with warmed medium prior to stimulation. 2D2 CD4^+^ T cells were isolated as described above and added to the microglia cultures along with the MOG_35-55_ peptide (25 μg/ml; Genescript) 4 hours after stimulation of the microglia.

### Cytokine and NO assays

Cytokine levels in the culture supernatants were measured by cytometric bead array (Th1Th2Th17 kit from BD Biosciences and Th1Th2Th17Th22 13-plex from eBioscience, San Diego, CA USA) or ELISA. All ELISA reagents were purchased from BD Biosciences with the exception of rIL-17 (eBioscience) and used according to manufactures instructions. NO production was measured in culture supernatants by Griess reaction as described [11].

### Flow cytometry

For analysis of cell surface markers, cells were incubated with Fc block (BD Bioscience) and subsequently incubated with the following fluorescently-labelled antibodies (all from BD Bioscience): rat anti-CD44, rat anti-CD25, rat anti-CD124, rat anti-CD62L, and rat anti-CD4. Background fluorescence was assessed using matched isotype control antibodies (BD Bioscience), and cells were analysed on a Canto II flow cytometer (BD Biosciences). Data was analysed using FlowJo 7.6.1 software (Tree Star, Ashland, OR, USA).

### Statistical analysis

All graphs and statistical analyses were generated using GraphPad Prism 5 (GraphPad Software Inc., La Jolla, CA USA). Comparisons between two groups were performed using a paired Student’s t test, and for non-normally distributed data, the non-parametric Wilcoxon matched pairs signed rank test was used. For comparison of more than two groups, a one-way ANOVA was used with a Neuman-Keuls’ multiple comparison post-test or a Dunnett’s multiple comparison test (to compare to a control condition). p < 0.05 was considered significant.

## Results

### Type II macrophages biased T cell responses and reduced T cell activation

**Fig 1:**
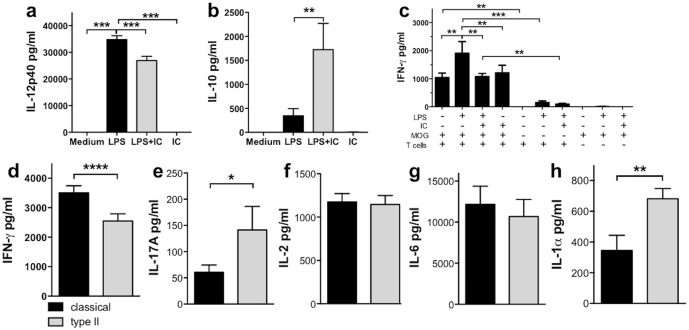
Type II macrophages expressed less IL-12 and more IL-10 than classical macrophages, and enhanced Th17 and Th2 biasing. (**a & b**) Macrophages were primed with IFN-γ (20 U/ml) overnight before stimulation with or without LPS (200 ng/ml) in the presence or absence of IC (10 IC/macrophage) for 24 hours. IL-12p40 and IL-10 levels were measured by ELISA. Shown are the means and SEM from 8 (**a**) or 10 (**b**) independent experiments. **p<0.01 and ***p<0.001 compared to LPS by one-way ANOVA with Dunnett’s multiple comparison test. (**c-h**) Type II macrophages biased the CD4^+^ T cell response to reduce Th1 and enhance Th17 cytokines. Macrophages were stimulated as described, and after four hours, purified CD4^+^ 2D2 T cells and MOG (25 μg/ml) were added and cultured for 72 hours. IFN-γ (**c & d**), IL-17A (**e**), and IL-2 (**f**) levels were measured by ELISA, and IL-6 and IL-1α levels by CBA (**g & h)**. Shown are the means and SEM from triplicate wells from one representative experiment (**c**) and the means and SEM from 11–15 (**d-f**) or 4 (**g & h**) independent experiments. *p<0.05, **p<0.01, ***p<0.001, and ****p<0.0001 by one-way ANOVA with Neuman-Keuls’ post-test (**c**) or paired Student’s t test (**d-h**).

Exposure of IFN-γ-primed macrophages to LPS causes classical activation and leads to a significant upregulation of IL-12 compared to IFN-γ-primed, unstimulated macrophages (i.e. medium alone; Fig 1a). Consistent with previous studies [3, 5], this production of IL-12 was significantly attenuated in IFN-γ-primed macrophages that were cultured with LPS in the presence of the type II activating stimulus, IC (Fig 1a). Additionally, macrophages incubated with LPS+IC had significantly increased levels of IL-10 compared to all other culture conditions (Fig 1b) while macrophages cultured with IC in the absence of LPS produced only low levels of IL-12 or IL-10 and were similar to macrophages cultured in medium alone (Fig 1a & 1b).

To determine how type II macrophages affected the biasing of CD4^+^ T cells, classical or type II macrophages were co-cultured with CD4^+^ T cells isolated from 2D2 mice, which express a MOG-specific TCR [12], and MOG_35-55_ peptide. Th biasing was determined 72 hours later by assessing IFN-γ (i.e.Th1) production, IL-17A (i.e. Th17), and the expression of CD124 (IL-4Rα; upregulated on Th2 cells). IFN-γ was used in the priming of macrophages; however, prior to macrophages stimulation, IFN-γ was removed from the cultures. Thus the IFN-γ in the supernatants was due to *in vitro* production, and not residual IFN-γ left over from the priming process. The production of the cytokines IFN-γ, IL-17A, and IL-2 was T cell dependent and MOG-specific as little or no cytokine was produced in the absence of CD4^+^ T cells or MOG (Fig 1c and Figure Ba-c in S1 File). CD4^+^ T cells activated by classical macrophages produced high levels of IFN-γ in an antigen-specific manner and expressed only low levels of IL-17A (Fig 1c–1e). Although IL-4 was not detected, CD124 was expressed on T cells cultured with unstimulated macrophages, and this level was reduced when T cells were stimulated by classical macrophages suggesting that classical macrophages bias CD4^+^ T cells away from a Th2 phenotype (Figure Bd in S1 File).

**Fig 2:**
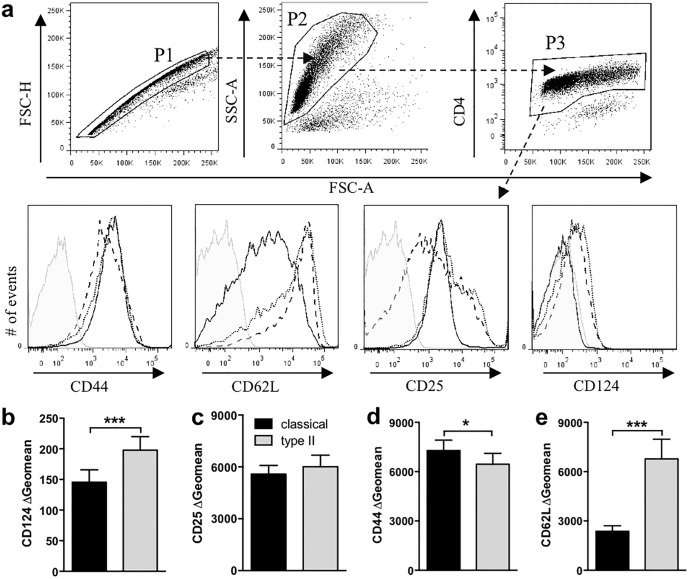
Type II macrophages reduced T cell activation markers. Macrophages were primed with IFN-γ (20 U/ml) overnight. IFN-γ was removed with warm media and the macrophages were stimulated with or without LPS (200 ng/ml) in the presence or absence of IC (10 IC/macrophage). After four hours, purified CD4^+^ 2D2 T cells and MOG (25 μg/ml) were added and cultured for 72 hours. CD124 (**b**), CD25 (**c**), CD44 (**d**), and CD62L (**e**) expression was assessed by flow cytometry. (**a**) Gating strategy to assess cell surface markers on T cells (P1, single cells; P2 live cells by FSC vs SSC; P3 CD4^+^ cells). Grey filled = Isotype control; solid line = classical macrophage; dotted line = type II macrophage; dashed line = unstimulated macrophage. (**b-e**) T cells cultured with type II macrophages express lower levels of CD124 (**b**) and higher levels of CD62L (**d**) compared to those cultured with classical macrophage. Shown are the geometric means from 17–19 individual experiments. *p<0.05 and ***p<0.001 by paired Student’s t test.

In contrast to cultures with classical macrophages, IFN-γ production by CD4^+^ T cells cultured with type II macrophages was reduced (Fig 1c and 1d). Interestingly, although IL-17A levels were low compared to IFN-γ levels, CD4^+^ T cells cultured with type II macrophages produced significantly more IL-17A than CD4^+^ T cells cultured with classical macrophages (Fig 1e). IL-2 levels were increased in both classical and type II macrophages cultures compared to unstimulated macrophages; however, IL-2 was present at similar levels in classical and type II macrophages cultures suggesting similar antigenic activation occurs in both culture conditions (Fig 1f and Figure Bb in S1 File). Similarly, IL-6 was increased to the same extent in both classical and type II macrophage cultures whereas IL-1α was significantly higher in type II macrophage containing cultures (Fig 1g & 1h). Additionally, CD4^+^ T cells cultured with type II macrophages expressed higher levels of CD124 (Fig 2a & 2b). Together these results indicate that antigen presented by type II macrophages may bias CD4^+^ T cells away from a Th1 phenotype and toward a mixed Th17/Th2 type response.

To explore further if altering the activation state of macrophages affected CD4^+^ T cell activation, the expression of CD25, CD62L, and CD44 was determined on CD4^+^ T cells co-cultured with classical or type II macrophages presenting MOG peptide. As with IL-2, similar levels of CD25 were detected on CD4^+^ T cells in these cultures suggesting a similar antigenic activation (Fig 2c). However, T cells cultured with type II macrophages had lower CD44 expression and increased CD62L expression compared to CD4^+^ T cells cultured with classical macrophages suggesting that classical macrophages are more effective at activating T cells (Fig 2c & 2d).

**Fig 3:**
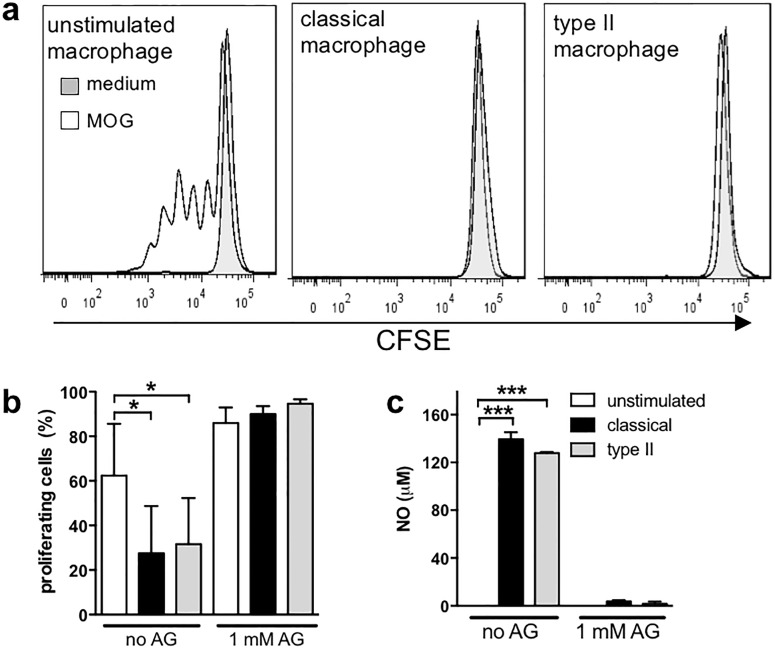
Type II macrophages do not enhance proliferation of CD4^+^ T cells in the absence of NO. Macrophages were stimulated as described in Fig 2 in the presence or absence of 1 mM aminoguanidine (AG). After four hours, purified CFSE-labeled CD4^+^ 2D2 T cells and MOG (25 μg/ml) were added to the cultures and cultured for 72 hours. Proliferation was analysed by flow cytometry, CD4^+^ T cells were gated as shown in Fig 2 and CFSE positive cells were gated as indicated using CFSE unlabeled cells in Fig 2. (**a**) Representative plots of CFSE staining from the different treatment conditions are shown (Solid line with MOG, filled line without MOG). (**b**) Percentage of cells that proliferated was calculated. (**c**) NO levels were measured by Griess reaction. Shown are the means and SEM combined from 3 independent experiments (**b**) or a representative experiment of 3 (**c**). *p<0.05 and ***p<0.001 by one-way ANOVA with Neuwman-Keuls’ post-test.

Because proliferation is the hallmark of an antigen-specific T cell response, MOG-specific T cell proliferation was assessed in the co-culture conditions using the CFSE dye dilution assay. Although there were antigen-specific T cell-derived cytokines produced in the co-culture system (Fig 1c and Figure B in S1a–S1c File), CD4^+^ T cells cultured with either classical or type II macrophages in the presence of MOG did not proliferate (Fig 3a & 3b). In contrast, unactivated macrophages were able to drive antigen-specific T cell proliferation effectively (Fig 3a & 3b). Since NO is known to be produced by classical macrophages and to inhibit T cell proliferation [13], NO levels in these cultures were assessed and found to be similarly and highly upregulated upon classical or type II activation (Fig 3c). In order to assess CD4^+^ T cell proliferation without the confounding effect of NO, aminoguanidine was added to the cultures to inhibit iNOS. The addition of aminoguanidine to the co-cultures significantly reduced NO and enabled T cell proliferation (Fig 3b & 3c). While CD4^+^ T cells cultured with classical or type II macrophages proliferated more in the presence of aminoguanidine, the increase in proliferation was similar (Fig 3b), suggesting that type II and classical macrophages have a similar ability to stimulate T cell proliferation.

### Enhanced Th17 cell biasing by type II activated macrophages does not involve IL-10, IL-12, or IL-6

**Fig 4:**
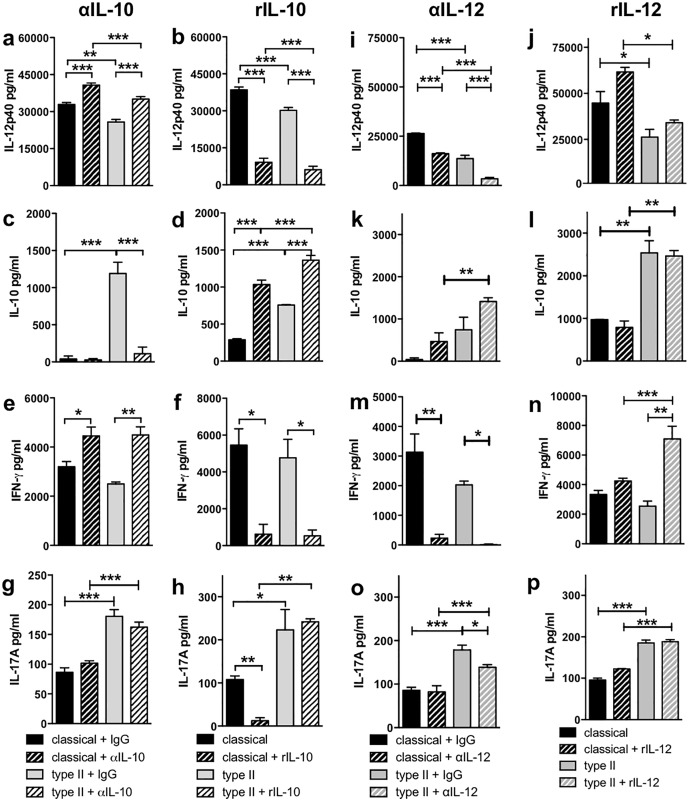
IL-10 and IL-12 regulated IFN-γ production but were not responsible for the enhanced Th17 biasing by type II macrophages. Macrophages were stimulated as described in Fig 2 in the presence or absence of αIL-10 (JES5-2A5, 2 μg/ml; **a, c, e, g**), rIL-10 (5 ng/ml; **b, d, f, h**), αIL-12 (C15.6, 2 μg/ml; **i, k, m, o**), rIL-12p70 (5 ng/ml; **j, l, n, p**), or isotype control (rat IgG_1_, 2 μg/ml; **a, c, e, g, i, k, m, o**). After four hours, purified CD4^+^ 2D2 T cells and MOG (25 μg/ml) were added and cultured for 72 hours. IL-12p40 (**a, b, i, j**), IL-10 (**c, d, k, l**), IFN-γ (**e, f, m, n**), and IL-17A (**g, h, o, p**) were assessed by ELISA. Shown are the means from 2–3 independent experiments (**a, c, b, f, h, k, l, m)** and means and SEM of triplicate wells from a representative experiment of at 2–5 independent experiments (**d, e, g, i, j, n, o, p)**. *p<0.05, **p<0.01, and ***p<0.001 by one-way ANOVA with Newman-Keuls’ post-test.

Previously IL-10 has been shown to be important in the type II macrophage mediated downregulation of the Th1 phenotype, but it is unknown whether IL-10 was involved in the enhanced Th17 biasing by type II macrophages. The addition of a blocking anti-IL-10 antibody (αIL-10) to classical or type II macrophage:T cell co-cultures led to a significant increase in IL-12 while the addition of rIL-10 decreased the production of IL-12 by both classical and type II macrophages when added at the time of macrophage stimulation (Fig 4a & 4b). As expected, neutralisation of IL-10 significantly increased IFN-γ levels produced by CD4^+^ T cells cultured with classical or type II macrophages (Fig 4e), and addition of rIL-10 resulted in the decreased production of IFN-γ from both CD4^+^ T cell co-cultures (Fig 4f). Interestingly, this decrease only occurred when rIL-10 was added to the co-cultures at the time of macrophage stimulation and not when the rIL-10 was added at the same time as the CD4^+^ T cells (Fig 4f and Figure Ca in S1 File). Despite altering IFN-γ production, αIL-10 did not significantly affect IL-17A production by CD4^+^ T cells co-cultured with classical or type II macrophages (Fig 4g) while exogenous rIL-10 reduced the level of IL-17A production in co-cultures containing classical macrophages but not type II macrophages (Fig 4h). Finally, CD124 and IL-2 expression were not significantly altered by either αIL-10 or rIL-10 (Figure Cb-e in S1 File). These results indicate that IL-10 production by type II macrophages may be in part responsible for the decreased production of IFN-γ by CD4^+^ T cells co-cultured with type II macrophages but does not account for the increased IL-17A production or CD124 expression.

IL-12 is the key cytokine driving Th1 differentiation and has been shown to reduce Th17 differentiation directly [14]. Thus, we investigated whether the reduced IL-12 produced by type II macrophages was responsible for the enhanced IL-17 production in the co-cultures. Neither neutralisation of IL-12 nor the addition of rIL-12 to macrophage:T cell cultures significantly altered IL-10 production (Fig 4k & 4l), whereas, as expected, neutralising IL-12 significantly decreased IFN-γ (Fig 4m). Interestingly addition of rIL-12 increased IFN-γ production only in co-cultures containing type II macrophages (Fig 4n) and suggests that the lower levels of IL-12 produced by type II macrophages may be at least partly responsible for the decreased level of IFN-γ produced by CD4^+^ T cells in these cultures. IL-17A levels were unchanged by altering IL-12 in classical macrophage cultures, and the addition of exogenous IL-12 did not alter IL-17A production in type II macrophages, but a significant decrease in IL-17A was observed when IL-12 was neutralized (Fig 4o & 4p). IL-2 levels and CD124 expression were unaltered when IL-12 was blocked or added (Figure D in S1 File). These data indicate that decreased IL-12 production by type II macrophages does not explain the enhanced CD124 or IL-17A expression by T cells.

**Fig 5:**
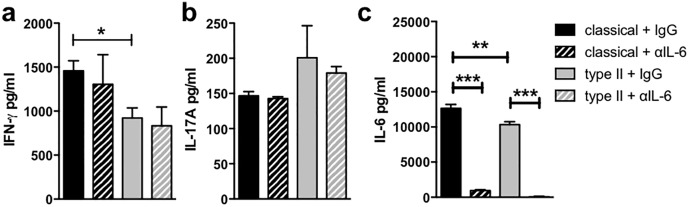
Neutralization of IL-6 did not prevent enhanced IL-17A production by CD4^+^ T cells cultured with type II macrophage. Macrophages were stimulated as described in Fig 2 in the presence of αIL-6 (MP5-20F3, 2 μg/ml) or isotype control (rat IgG_1_, 2 μg/ml). After four hours, purified CD4^+^ 2D2 T cells and MOG_35-55_ (25 μg/ml) were added to the macrophage cultures for 72 hours. IFN-γ (**a**), IL-17A (**b**), and IL-6 (**c**) were measured by ELISA. Shown are the means from 2 independent experiments (**c)** and means and SEM of triplicate wells from a representative experiment of 2–3 independent experiments (**a & b)**. *p<0.05, **p<0.01, and ***p<0.001 by one-way ANOVA with Newman-Keuls’ post-test.

Because the traditional pathway by which Th17 cells are induced is through exposure to IL-6 in the presence of TGF-β and very high levels of IL-6 were produced in this co-culture system (Fig 1g), the role of IL-6 in T cell biasing by type II macrophages was investigated. Although IL-6 was effectively neutralized in the co-cultures with type II and classical macrophages, the absence of IL-6 did not significantly change any of the markers of T cell biasing measured in this study (Fig 5a–5c and Figure E in S1 File). In particular, no significant change in IL-17A was seen, suggesting that it is not the IL-6 pathway that is driving the enhanced IL-17A production in the type II macrophage:T cell co-cultures.

### Microglia can be type II activated in vitro and bias T cell responses

**Fig 6:**
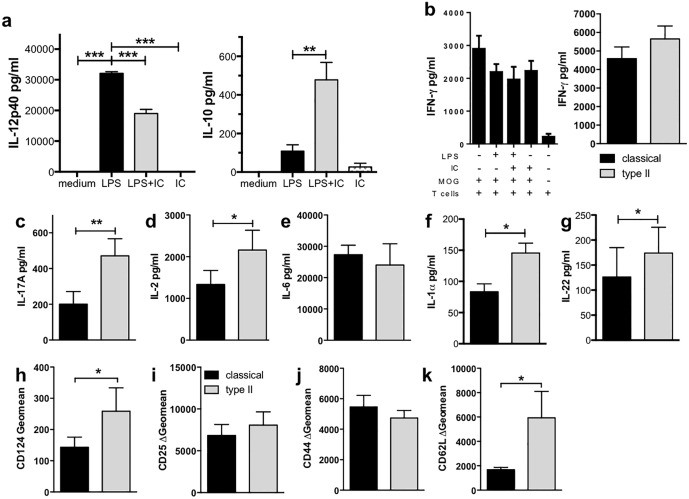
Type II microglia biased CD4^+^ T cell responses to a mixed Th17/Th2 phenotype. (**a**) Microglia can be type II-activated. Microglia were isolated from the CNS of adult mice (n = 5/experiment) and expanded in the presence of M-CSF (5 ng/ml) for 4 weeks. Microglia were primed with IFN-γ (20 U/ml) overnight before stimulation with LPS (200 ng/ml) with or without IC (10^6^/well) for 24 hours. Shown are the means and SEM of triplicate wells from a representative experiment of 3 independent experiments. **p<0.01 and ***p<0.001 by one-way ANOVA with Newman-Keuls’ post-test. (**b-k**) Type II microglia enhanced IL-17A production by CD4^+^ T cells. Microglia were isolated and activated as described. After four hours, purified CD4^+^ 2D2 T cells and MOG_35-55_ (25 μg/ml) were added for 72 hours. IFN-γ (**b**), IL-17A (**c**), IL-2 (**d**), and IL-6 (**e**) levels were measured by ELISA and IL-1α (**f**) and IL-22 (**g**) levels by CBA. CD124 (**h**), CD25 (**i**), CD44 (**j**), and CD62L (**k**) were assessed by flow cytometry. Shown are the means 7 (**b-d, h-k**) or 3 (**e & g**) independent experiments or the means and SEM of triplicate wells from a representative experiment of 3 independent experiments (**b & f**). *p<0.05, **p<0.01, and ***p<0.001 by one-way ANOVA with Newman-Keuls’ post-test (**a**) or Wilcoxon matched pairs signed rank test (**b-k**).

Microglia are important in the regulation of neuroinflammatory conditions, and activation of microglia to a regulatory phenotype similar to type II macrophages may be protective in conditions where neuroinflammation causes damage to the CNS. Thus, the ability of microglia to be type II activated and differently activate and bias T cell responses was investigated. As shown previously [15, 16], microglia significantly upregulated IL-12 production when stimulated with LPS (Fig 6a). In contrast, when microglia were stimulated with LPS in the presence of IC (i.e. type II-activated), the microglia produced significantly less IL-12 and more IL-10 compared to LPS alone (Fig 6a). Microglia cultured in medium alone or IC produced only low levels of IL-12 and IL-10 (Fig 6a). This pattern of cytokine expression in LPS+IC-treated microglia is suggestive of type II activation, and the changes are consistent with those seen in type II macrophages.

To determine if type II microglia could differentially activate and bias naïve CD4^+^ T cells, the co-culture system using MOG and 2D2 CD4^+^ T cells was used. Microglia were able to drive antigen-specific T cell responses under non-biasing conditions (i.e. unstimulated microglia) as demonstrated by increased IFN-γ and IL-2 compared to cultures in which MOG was absent (Figure F in S1 File). As with macrophage cultures, IFN-γ was removed from the microglia culture prior to stimulation, therefore, IFN-γ detected was due to *in vitro* production. Interestingly, in this system, CD4^+^ T cells produced a high level of IFN-γ regardless of the microglia activation state (Fig 6b), and this finding is in stark contrast to macrophage:T cell co-cultures (Fig 1c). As observed with the macrophage:T cell co-cultures, CD4^+^ T cells cultured with type II microglia produced significantly more IL-17A compared to co-cultures with classical or unstimulated microglia (Fig 6c), and this enhanced IL-17A was not driven by IL-6 as neither neutralization of IL-6 in the type II microglia:T cell co-cultures nor addition of exogenous IL-6 to classical microglia:T cell cultures affected IL-17A production (Figure G in S1 File). CD4^+^ T cells cultured with type II microglia produced significantly more IL-2, IL-1α, and IL-22 and had an increased expression of CD124 (Fig 6d, 6f, 6g & 6h). Finally, CD4^+^ T cells cultured with classical or type II microglia expressed a similar pattern as classical and type II macrophages with an increased expression of CD62L and CD124 on type II microglia (Fig 6h & 6k). Overall, these data suggest that type II microglia activate CD4^+^ T cells in a manner that is similar but not identical to type II macrophages.

### T cell biasing by type II macrophages induced by different type II activating treatments

**Fig 7:**
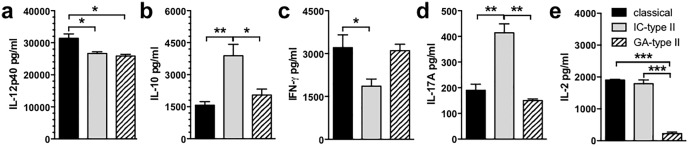
GA-type II macrophages and IC-type II macrophages have different effects on naïve CD4^+^ T cells. Macrophages were stimulated with IFN-γ (20 U/ml) overnight. IFN-γ was removed with warm media and the macrophages were then cultured with LPS (200 ng/ml) in the presence or absence of IC (10/macrophage) or GA (100 μg/ml). Four hours after macrophage activation, purified CD4^+^ 2D2 T cells and MOG_35-55_ (25 μg/ml) were added to the macrophage cultures for 72 hours. (**a-e**) All cytokines were measured by ELISA. Shown are the means and SEM of triplicate wells from a representative experiment of 3–8 independent experiments. *p<0.05, **p<0.01, and ***p<0.001 by one-way ANOVA with Newman-Keuls’ post-test.

Type II macrophages are broadly characterised as producing increased IL-10 and decreased IL-12, and several compounds have been shown to induce a type II activation state including IC, glatiramer acetate (GA), prostaglandins, and inhibitors of salt-inducible kinases [2, 17, 18]. However, while these activation states share many common traits, type II macrophages induced through different pathways do not necessarily represent identical phenotypes. To investigate this possibility, we compared the effect of type II macrophages generated by LPS+IC (IC-type II macrophages) and LPS+GA (GA-type II macrophages) on CD4^+^ T cell biasing to understand if the enhanced IL-17A production was an outcome common to another type II-activating compound. GA-type II macrophages produced decreased levels of IL-12 compared to classical macrophages and similar to that seen by IC-type II macrophages (Fig 7a). GA-type II macrophages produced slightly enhanced levels of IL-10, but not to the same extent as IC-type II macrophages (Fig 7b). GA-type II macrophages were efficient antigen presenting cells and induced CD4^+^ T cells to produce a similar level of IFN-γ as those cultured with classical macrophages (Fig 7c). Interestingly, GA-type II macrophages were not as efficient at inducing IL-17A or IL-2 production as IC-type II macrophages (Fig 7d & 7e). Taken together, these data suggest that type II macrophages induced by different type II activation pathways are distinctly different, and this difference may alter functional outcomes when macrophages interact with CD4^+^ T cells.

## Discussion

This study explored and compared how type II activation of macrophages and microglia directed antigen-specific naïve CD4^+^ T cell responses. Consistent with previous reports [5] classical macrophages induced a Th1 response from naïve CD4^+^ T cells characterised by high levels of IFN-γ, and CD4^+^ T cells cultured with type II macrophages produced lower levels of IFN-γ. Interestingly, CD4^+^ T cells cultured with type II macrophages or type II microglia expressed more CD124 and IL-17A suggesting that type II macrophages and type II microglia bias the T cell response towards a mixed Th2/Th17 phenotype, but this shift was not mediated by IL-10, IL-12, or IL-6 (Table 1). Finally, while the most distinct effect on T cell responses induced by type II macrophages and type II microglia was enhanced IL-17A production, type II activation by another type II-activating compound, GA, did not induce a similar increase in IL-17A production. Together our results indicate that even within the type II activated subset, a wide spectrum of functional states and phenotypes exist.

**Table 1:** IL-10 and IL-12 are key regulators of IFN-γ in macrophage: T cell cultures while IL-6 does not contribute significantly to T cell biasing in this system.

		Classical macrophages	Type II macrophages
IL-10	Neutralise	↑IFN-γ, ↑IL-12	↑IFN-γ, ↑IL-12
	rIL-10	↓IFN-γ, ↓IL-12, ↓IL-17A	↓IFN-γ, ↓IL-12
IL-12	Neutralise	↓IFN-γ	↓IFN-γ, ↓IL-17A
	rIL-12	No change	↑IFN-γ
IL-6	Neutralise	No change	No change
	rIL-6	No change	No change

IL-10 is an anti-inflammatory cytokine known to reduce the production of IFN-γ by T cells and has been implicated in T cell biasing by type II macrophages [5]. In agreement with Anderson *et al* [5], we found that IL-10 production by type II macrophages was a key regulator of IFN-γ production. IL-10 mediates the majority of its effects by inducing downregulation of inflammatory cytokines, such as IL-12, and co-stimulatory molecules on antigen presenting cells (APC), resulting in downstream effects on the T cells or other APC [19–22]. Previous studies have shown that IL-10 can directly act on T cells to inhibit the production of cytokines [23–25]; however, our results indicate that the ability of IL-10 to reduce IFN-γ production was mediated by effects on the APC given that the addition of IL-10 was only effective before the addition of T cells. Because blocking IL-10 significantly increased IL-12 production by type II macrophages but the addition or neutralization of IL-12 did not affect IL-10 levels, we believe that the enhanced IL-10 production by type II macrophages contributes to the reduced IL-12. IL-10 is known to inhibit IL-12 production [22, 25], and as IL-12 is a key cytokine in the induction of Th1 cells, the reduction of IFN-γ is to be expected. These results suggest that the IL-10 regulates IFN-γ production by reducing IL-12 production by type II macrophages.

As shown previously, IFN-γ was significantly reduced in macrophage:T cell co-cultures containing type II macrophages; however, while significant, the decrease in IFN-γ in our study was less marked than what has been observed previously [4, 5]. Additionally, we did not detect any IL-4 in the macrophage:T cell co-cultures in contrast to previous reports [4, 5], and instead, we used the expression of CD124 as a marker of Th2 responsiveness. Several differences in our co-culture systems may account for the observed variations in IFN-γ and IL-4 expression such as mouse strain and TCR specificities. Previous work on T cell biasing by type II macrophages employed mice on a BALB/c background [4, 5], whereas the macrophages and T cells from the current study were from a C57BL/6 background. Because BALB/c mice preferentially produce a Th2 response, it is likely the T cells used in previous studies (BALB/c background) were more susceptible to Th2 biasing conditions than the ones used in this study (C57BL/6). Similarly, because C57BL/6 mice preferentially produce strong Th1 responses, type II activation may be less effective at reducing these responses. Finally, our study used the MOG-specific 2D2 TCR in contrast to the OVA-specific DO.11.10 TCRαβ [5]. As it is known that the strength of TCR signalling can affect T cell biasing, it is also possible that different T cell clones with different signal strengths may be predisposed to bias T cells to a specific subset [26, 27].

This study is the first to show that type II macrophages and type II microglia can enhance Th17 biasing. Both IL-12 and IFN-γ inhibit IL-17A production [14, 28] suggesting that the decreases in IL-12 and IFN-γ may have enabled the increase in IL-17A. However, the addition or neutralization of IL-12 had no effect on IL-17A production despite significantly altering IFN-γ and thus Th1 biasing in classical macrophage cultures. Addition of rIL-12 also had no effect on IL-17A levels in type II macrophage cultures, however a modest but significant decrease in IL-17A type II macrophage cultures was found when IL-12 was neutralized. As IL-12 is inhibitory to IL-17A production, neutralization of IL-12 would be expected to drive IL-17A production, therefore this change does not suggest that the lower IL-12 produced by type II macrophages is responsible for the increased IL-17A production. While IL-10 was shown to be the main regulator of Th1 biasing in our co-culture system, it was not found to be responsible for promoting Th17. Given that IL-10 suppresses IL-17A production [29], this result is not surprising. Indeed, as expected, the addition of IL-10 to classical macrophage:T cell co-cultures abolished IL-17A production. In contrast, the addition of exogenous IL-10 to type II macrophage:T cell co-cultures did not inhibit IL-17A further supporting that the pathways driving T cell biasing by classical macrophages and type II macrophages are distinct.

In mice, the presence of IL-6 with TGF-β represents the predominant pathway driving Th17 development [30]. IL-6 was present in high levels in macrophage:T cell cultures containing both classical and type II macrophages but we found that neutralization of IL-6 had no effect on IL-17A in cultures containing classical or type II macrophages. Furthermore, this finding was also confirmed in microglia:T cell co-cultures. Given that in the absence of IL-6, TGF-β induces T regulatory cells not Th17 [30], these findings indicate that IL-17A production is driven by another pathway in this system.

One of the key findings of this study was that both type II macrophages and type II microglia promote increased IL-17A from naïve CD4^+^ T cells via an IL-6-independent pathway, and no other pathway investigated in macrophages:T cell co-cultures could account for the increase in IL-17A induced by type II macrophages. While IL-6/TGF-β is considered the classical pathway for Th17 development, other pathways have been reported. In particular, IL-1α in conjunction with IL-23 has been shown to induce Th17 cells [31], and co-cultures containing type II macrophages or type II microglia had significantly more IL-1α than classical macrophages or classical microglia co-cultures suggesting that IL-1α represents a possible pathway enhancing IL-17A production. However, given that the neutralization of the IL-12p40 subunit, which is shared by IL-23, did not significantly reduce IL-17A production, this pathway is unlikely. Finally, as it has been reported previously that Th17 cells have a transient nature and can further differentiate into other Th subsets [32, 33], an alternative explanation for our findings is that the IL-17A producing cells may be in transition to another Th subset.

Evidence suggests that interaction of invading cells with local CNS cells such as microglia is important in the development of CNS immune responses such as those that occur in EAE [7]. Thus microglia have great potential to influence T cell responses and affect disease outcome. Our findings indicate that classical, type II, and even unactivated microglia were equally capable of driving Th1 responses, possibly due to the presence of low levels of IFN-γ during priming. These high levels of IFN-γ produced in the co-culture conditions were despite the significantly different levels of IL-12 in cultures with LPS-stimulated and non-stimulated microglia cultures and is in contrast to the macrophages co-cultures, where classical macrophages and type II macrophages induced T cells to produce significantly more IFN-γ than unstimulated macrophages. In the current study, relatively low concentrations of IFN-γ (20 U/ml) were used to prime both macrophages and microglia, and IFN-γ doses as low as 10 U/ml have been shown to stimulate microglia to become efficient APC to pre-activated Th1 and Th2 cell lines [34]. It is possible that microglia are more sensitive to IFN-γ due to their residence in an immune privileged environment where they would not be readily exposed to IFN-γ. By comparison, peripheral macrophages, which are more commonly exposed to IFN-γ, may have a higher threshold for IFN-γ stimulation to prevent aberrant activation and collateral tissue damage.

Type II microglia bias the T cell response in a distinct way from type II macrophages, although some features are shared. For example, both increase the expression of IL-17A and CD124 on T cells. The biasing of T cells toward Th17 by type II microglia would potentially be detrimental in neuroinflammatory conditions as Th17 responses are considered pathogenic in EAE and are associated with the recruitment of neutrophils capable of causing significant tissue damage [35]. That said, it should be noted that the level of IL-17A production, while significantly increased in T cells cultures with type II microglia compared to other conditions, is relatively low compared to IFN-γ, therefore the functional consequence of the increased IL-17A *in vivo* remains to be determined.

Type II macrophages can be induced through exposure to a variety of compounds including IC, GA, prostaglandins, and inhibitors of salt inducible kinases [2, 17, 18]. GA, a common drug for the treatment of MS, has been shown to induce type II activation of monocytes as indicated by a shift in the IL-10/IL-12 profile and an ability to bias T cell responses towards Th2 *in vivo* [18]. However, in contrast to IC, we found that GA did not induce an increase in IL-17A or IL-2 production from T cells. This finding is in agreement with previous work showing that GA decreases the production of IL-17A in the periphery and in the CNS of mice with EAE [18, 36]. As GA and IC appear to activate macrophages to type II activation through different mechanisms, how these two type II-activating compounds interact merits investigation.

In conclusion, this study demonstrated that both macrophages and microglia can be type II activated and can modify T cell responses by biasing T cells toward to mixed Th17/Th2 phenotype. This study is the first to show that type II macrophages and type II microglia promote IL-17A production by T cells and that this pathway is independent of IL-10, IL-12, and IL-6. The enhanced IL-17A is especially counterintuitive given that type II macrophages are protective in EAE. However, while the increase in IL-17A is observed with IC- type II macrophages, GA- type II macrophages do not induce the same increase in IL-17A but maintain a similar IL-10/IL-12 profile. Thus, despite the increase in IL-17A by IC-mediated type II activation, type II macrophages and type II microglia activated by other type II compounds likely represent a protective subset of cells, which, if induced *in vivo* may help protect against inflammatory conditions such as MS.

## Supporting Information

S1 File Figures A-G. (DOCX)
