# Supplementary material for: Type II Activation of Macrophages and Microglia by Immune Complexes Enhances Th17 Biasing in an IL-6-Independent Manner
Source: PLoS One. 2016 Oct 12;11(10):e0164454. doi: 10.1371/journal.pone.0164454 (PMC5061352; doi:10.1371/journal.pone.0164454)
Supplement: S1 File — (DOCX) [file pone.0164454.s001.docx]

**Supplementary Figures**


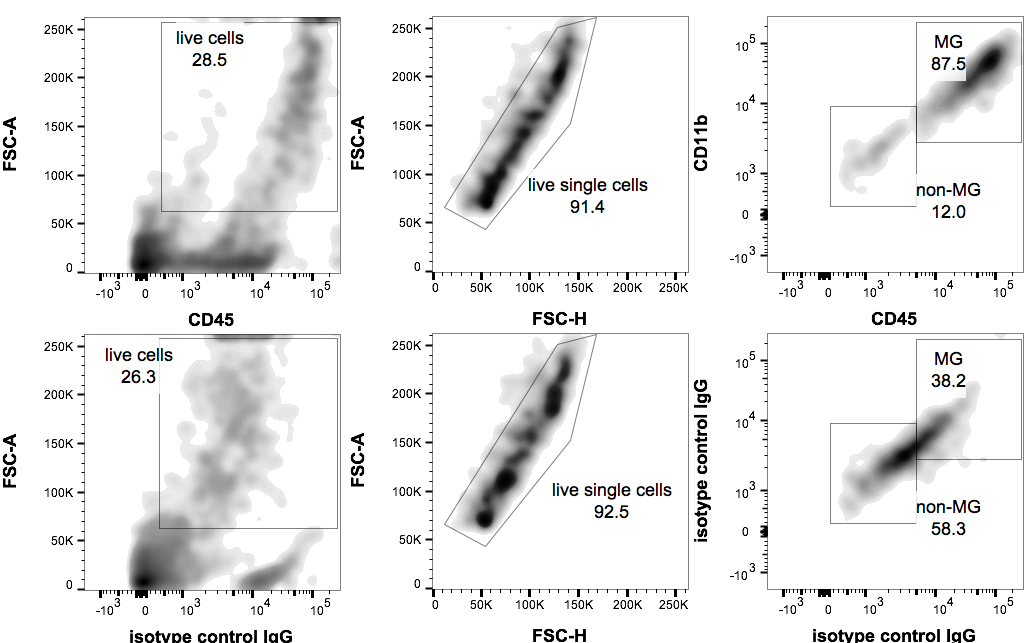


Supplementary Figure A: Microglial cultures are highly pure after 4 weeks in culture. Microglia were isolated from the CNS of adult mice (n=5) and plated at 5x10^4^ cells/well in the presence of M-CSF (5 ng/ml). After four weeks in culture microglia purity was assessed by flow cytometry using CD45 and CD11b. Shown are representative plots from one experiment.

a.

c.

b.

d.

Supplementary Figure B: Type II macrophages alter T cell responses. Macrophages were primed with IFN-γ overnight before stimulation with or without LPS (200 ng/ml) in the presence or absence of IC. After four hours, purified CD4^+^2D2 T cells and MOG (25 μg/ml) were added and cultured for 72 hours. IL-17A (a) and IL-2 (b) levels were measured by ELISA, IL-6 levels (c) were measured by CBA. CD124 levels (d) were measured by flow cytometry. Shown are the means and SEM of one representative experiment from at least 3 individual experiments

**a**

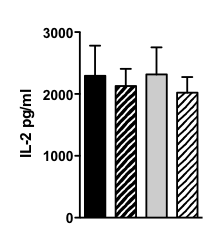

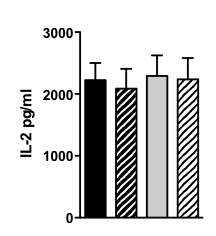


**b**

**d**

**c**

**e**

Supplementary Figure C: Changes in IL-10 do not fully explain the T cell biasing by type II macrophages. Macrophages were stimulated as described. rIL-10 (5 ng/ml), αIL-10 (JES5-2A5, 2 μg/ml) or rat isotype (IgG_1_, 2 μg/ml) were added at the time of macrophages stimulation (**a-e**) or T cell addition (four hours, **a**). Four hours after stimulation, purified CD4^+^2D2 T cells and MOG (25 μg/ml) were added and cultured for 72 hours. IFN-γ and IL-2 were assessed by ELISA and CD124 expression was measured by flow cytometry. Shown are the means and SEM of triplicate wells from at least 3 experiments. *p<0.05 by one-way ANOVA with Newman-Keuls’ post-test.

Classical

macrophages

Type II

macrophages

Supplementary Figure D: Changes in IL-12 does not strongly affect IL-2 or CD124 levels in macrophage:T cell co-cultures. Macrophages were stimulated as described in the presence or absence of rIL-12p70 (5 ng/ml), αIL‑12 (C15.6, 2 μg/ml) or rat isotype (IgG_1_, 2 μg/ml). After four hours, purified CD4^+^2D2T cells and MOG_35-55_ (25 μg/ml) were added to the macrophage cultures for 72 hours. IL-2 was measured by ELISA and CD124 was measured but flow cytometry. Shown are the means and SEM of triplicate wells from at least 2 experiments, IL-12 and IL-17A data is presented as a percentage of “classical macrophages with no antibody/protein”. *p<0.05, **p<0.01, and ****p<0.0001 by Student’s t test.

Classical

macrophages

Type II

macrophages

Supplementary Figure E: Altering IL-6 levels in macrophage:T cell co-culture does not affect IL-12p40, IL-10, IL-2 or CD124 levels. Macrophages were stimulated with IFN-γ overnight and then cultured with LPS (200 ng/ml) alone or with IC (10 per macrophage). rIL-6 (5 ng/ml), αIL‑6 (MP5-20F3, 2 μg/ml) or rat isotype (IgG_1_, 2 μg/ml) was added to cultures with the stimuli. After four hours, purified CD4^+^2D2 T cells and MOG_35-55_ (25 μg/ml) were added to the macrophage cultures for 72 hours. IL-12, IL‑10, and IL-2 were measured by ELISA, CD124 was measured by flow cytometry. Shown are the means and SEM of triplicate wells from at least 2 experiments. ****p<0.0001 by Student’s t test.

Supplementary Figure F: Under non-biasing conditions microglia can present to T cells and drive an antigen specific response. Microglia were isolated from the CNS of adult mice (n=5) and plated at 5x10^4^ cells/well in the presence of M-CSF (5 ng/ml). After four weeks in culture M-CSF was removed and microglia were primed with IFN-γ overnight before purified CD4^+^2D2 T cells were added to the microglia cultures with or without MOG_35-55_ (25 μg/ml), cells were cultured for 72 hours. IFN‑γ and IL-2 levels were measured by ELISA. Shown are the mean and SEM of at least duplicate wells from two combined experiments. **p<0.01, ***p<0.001 by unpaired student’s t test.

Classical

microglia

Type II

microglia

Classical

microglia

Type II

microglia

Supplementary Figure G: Altering IL-6 levels in microglia cell co-culture is not responsible for increased IL-17A and does not alter any other pathway. Microglia were isolated from the CNS of adult mice (n=5) and plated at 5x10^4^ cells/well in a flat bottomed 96 well plate in the presence of M-CSF (5 ng/ml). After four weeks in culture M-CSF was removed and microglia were primed with IFN-γ overnight before stimulation with LPS (200 ng/ml) alone or with IC. rIL-6 (5 ng/ml), αIL‑6 (MP5-20F3, 2 μg/ml) or rat isotype (IgG_1_, 2 μg/ml) was added to cultures with the stimuli. After four hours, purified CD4^+^2D2 T cells and MOG_35-55_ (25 μg/ml) were added to the microglia cultures for 72 hours. After four hours, purified CD4^+^2D2 T cells and MOG_35-55_ (25 μg/ml) were added to the microglia cultures for 72 hours. . IFN-γ, IL‑17A, IL-6, IL-12p40, IL‑10, and IL-2 were measured by ELISA. Shown are the means and SEM of triplicate wells from 2-3 experiments. *p<0.05, *p<0.01, ****p<0.0001 by student’s t test.
